# Supplementary material for: Development of culturally-appropriate text message booster content to follow a brief intervention focused on reducing alcohol related harms for injury patients in Moshi, Tanzania
Source: PLOS Glob Public Health. 2024 Jul 25;4(7):e0002717. doi: 10.1371/journal.pgph.0002717 (PMC11271911; doi:10.1371/journal.pgph.0002717)
Supplement: S1 Protocol — (DOC) [file pgph.0002717.s005.doc]

# Title Page

**Developing a Brief Negotiational Intervention for Alcohol in Tanzania**

US Principal Investigator:

Catherine A. Staton, MD MScGH

Assistant Professor of Surgery

Division of Emergency Medicine

Duke Global Health Institute

[catherine.Staton@duke.edu](mailto:catherine.lynch@duke.edu)

US cell (908) 872-9208

Tanzanian Principal Investigator

Blandina Mmbaga

Director, KCMC/Duke Collaboration

Kilimanjaro Christian Medical Center

[blaymt@gmail.com](mailto:blaymt@gmail.com)

Other Investigators

Mark Mvungi, MD

Director, Casualty Department

Kilimanjaro Christian Medical Center

mvungigloria@yahoo.co.uk

Table of Contents

[Title Page 1](#__RefHeading___Toc424008197)

[Abstract: 3](#__RefHeading___Toc424008198)

[Abbreviations: 3](#__RefHeading___Toc424008199)

[Introduction: 4](#__RefHeading___Toc424008200)

[Literature Review: 5](#__RefHeading___Toc424008201)

[Injuries 5](#__RefHeading___Toc424008202)

[Alcohol 5](#__RefHeading___Toc424008203)

[Brief Negotiational Intervention 5](#__RefHeading___Toc424008204)

[Statement of the Problem: 6](#__RefHeading___Toc424008205)

[Rationale: 7](#__RefHeading___Toc424008206)

[Broad Objectives: 7](#__RefHeading___Toc424008207)

[Specific Objectives: 7](#__RefHeading___Toc424008208)

[Methods: 7](#__RefHeading___Toc424008209)

[Aim 1: Identify patients, families, healthcare providers’ and the public’s current perspectives on alcohol use. 8](#__RefHeading___Toc424008210)

[Study Design: 8](#__RefHeading___Toc424008211)

[Study Population: 10](#__RefHeading___Toc424008212)

[Inclusion/ Exclusion Criteria: 10](#__RefHeading___Toc424008213)

[Sample Size Calculation: 11](#__RefHeading___Toc424008214)

[Data Collection: 11](#__RefHeading___Toc424008215)

[Data Analysis: 11](#__RefHeading___Toc424008216)

[Aim 2: Develop a culturally- and linguistically-appropriate, nurse-delivered BNI; validate measures to test its effectiveness 12](#__RefHeading___Toc424008217)

[Summary: 12](#__RefHeading___Toc424008218)

[Validation of Alcohol Outcome Scales: 13](#__RefHeading___Toc424008219)

[Aim 3: Conduct a pilot trial of the intervention. 14](#__RefHeading___Toc424008220)

[Introduction and Rationale: 14](#__RefHeading___Toc424008221)

[Research Design: 14](#__RefHeading___Toc424008222)

[Participants and Recruitment: 14](#__RefHeading___Toc424008223)

[Follow up Procedures: 15](#__RefHeading___Toc424008224)

[Feasibility Trial Outcomes 15](#__RefHeading___Toc424008225)

[Sample Size 16](#__RefHeading___Toc424008226)

[References: 18](#__RefHeading___Toc424008227)

# Abstract:

Annually, there are 1.8 million global deaths due to alcohol. Alcohol use is rapidly increasing in low and middle-income countries, where it is inexpensive, readily available, poorly regulated, and there are few resources devoted to safe alcohol use behavior. Like many other limited resource settings, there are few treatment facilities or addiction practitioners in the Kilimanjaro region of Tanzania. This project allow for the cultural adaptation, validation, and pilot testing of a brief negotiational interview for alcohol use. To do so, the project will 1) describe the current knowledge and perceptions about alcohol use in Tanzania, 2) adapt an evidence-based intervention to the Tanzanian setting and, 3) pilot this intervention in preparation for a large-scale trial. The intervention, a brief negotiational interview, based on a motivational interviewing framework, has proven effective in the US but has not been adapted or implemented in an African context.

# Abbreviations:

BNI brief negotiational interview

CAB Community Advisory Board

DALY disability-adjusted life-years

LMIC low and middle income countries

SBI screening and brief intervention

WHO WMH-CIDI World Health Organization World Mental Health Composite International Diagnostic Interview.

aaPDD alcohol adapted Perceived Devaluation Scale

# Introduction:

Violence and injuries are a global health crisis. Almost 6 million people die annually from, or related to their injuries.^1^ Over 90% of unintentional injuries and 90% of disability-adjusted life-years (DALYs) lost occur in low and middle income countries (LMIC).^1^, In most countries around the world, a large proportion of adults consume alcohol. Globally, alcohol causes 3.2% of all deaths or 1.8 million deaths annually and accounts for 4.0% of the disease burden.^2^ Many of these deaths are the result of injuries caused by hazardous and harmful drinking.^2^ Substance use has been shown to not only increase injury risk and severity but also to worsen outcome.^3-14^ Just as injury disproportionally affects LMIC, alcohol-related injuries are particularly concerning in LMIC where consumption rates are increasing, and public health infrastructure is not equipped to curb these injuries.^2^

In high-income settings, secondary prevention initiatives such as a screening and brief intervention (SBI) administered by healthcare practitioners has been demonstrated in primary healthcare settings to reduce a patient’s alcohol intake for up to two years post-intervention and has been proven to be cost-effective.^15^ ^16^ ^17^ SBI have also been shown to be efficacious for patients who suffered alcohol related injuries in the acute setting, leading to a decrease in overall post injury alcohol use at one year post injury as well as a 48% reduction in injury recurrence rate within 3 years post injury.^18^ While SBI has not been validated in LMIC, understanding current practitioner knowledge, attitudes and practices on alcohol use disorders, and alcohol related injuries will assist in developing a culturally appropriate and resource feasible SBI.

# Literature Review:

### Injuries

Violence and injuries are a global health crisis. Almost 6 million people die annually from, or related to their injuries.^1^ Overall, injuries account for about 10% of the world’s deaths.^1^ While injuries affect all age groups, they have a particular impact on young people especially those between 5 and 44 years of age.^1^ Road traffic injuries are one of the top three causes of death for people between 5 and 44 years of age.^1^ Injuries are not only a leading cause of mortality but also morbidity. In addition to the 6 million people who die annually, there are approximately 650 million people worldwide who are living with disabilities due to injury which accounted for about 138 million disability-adjusted life-years (DALYs) lost in 2004 alone.^1,19^ Over 90% of unintentional injuries and 90% of DALYs occur in low and middle income countries (LMIC).^1,19^ The highest burden of injury mortality and morbidity occurs in LMIC and coincides with the greatest challenges providing care, the weakest evidence base to guide interventions, and the fewest resources and infrastructure to institute effective change.

### Alcohol

In most countries around the world, a large proportion of adults consume alcohol. Alcohol is associated with adverse consequences for the drinker and society at large. Globally, alcohol causes 3.2% of all deaths or 1.8 million deaths annually and accounts for 4.0% of the disease burden.^2^ Many of these deaths are the result of injuries caused by hazardous and harmful drinking.^2^ Of the total number of alcohol attributable deaths, 32% are from unintentional injuries and 13.7% are from intentional injuries.^2^ Overall, half of all deaths attributable to alcohol are due to injuries. Some studies have estimated the proportion of injuries with alcohol involvement between 6% and 45% depending on the country.^2^ Substance use has been shown to not only increase injury risk and severity but also to worsen outcome.^3-14^ Just as injury disproportionally affects LMIC, alcohol-related injuries are particularly concerning in LMIC where consumption rates are increasing, and public health infrastructure is not equipped to curb these injuries.^2^ Collection of accurate data on alcohol and injuries at hospitals is vital to understanding both the scope and nature of the problem specifically in Tanzania.

Brief Negotiational Intervention.

Since patients in Emergency Departments seeking care for alcohol related injuries have been shown to have continued alcohol use, increased impaired driving and an increased mortality rate^20^, they are those that should receive an intervention to reduce their harmful alcohol use. A brief negotiational intervention (BNI) administered in an Emergency Department (ED) setting

has been shown to cost-effectively reduce a patient’s alcohol intake for up to two years post-intervention.^15-17,21,22^ A BNI are short, 5-60 minutes counseling sessions administered by non-

addiction specialists based on the concepts of motivational interviewing.^21^ The FRAMES model for motivational interviewing (Table 1)

| **FRAMES Model** | |
| --- | --- |
| **Feedback** | Give feedback on the risks and negative consequences of substance use.  Seek the client's reaction and listen |
| **Responsibility** | Emphasize the individual’s responsibility for making his/ her own decision about his/her alcohol use |
| **Advice** | Give straightforward advice on modifying alcohol use |
| **Menu of options** | Give menu of options to choose from, fostering the client's involvement in decision-making |
| **Empathy** | Be empathetic, respectful and non-judgmental. |
| **Self-Efficacy** | Express optimism about his/her ability to modify his/her substance abuse. |
| Table 1: FRAMES Model | |

is a well-accepted framework whereby a provider and patient discuss alcohol use in a non-judgmental manner, where the patient establishes goals of alcohol reduction after accepting responsibility for his/her actions.^23^ BNIs are successful due to their non-judgmental, informative motivational nature infused with both information about alcohol use consequences and self-determined goals of substance use reduction that increase self-efficacy. The 1990 landmark Institute of Medicine report recommended brief interventions based on a motivational interviewing framework aimed at mild to moderate alcohol use given the sheer strength of the literature supporting brief interventions.^24^ ^25^ In the late 1990s, admitted United States trauma patients who received an alcohol BNI at discharge were shown to have a decrease in overall post injury alcohol use at one year post injury as well as a 48% reduction in injury recurrence rate within 3 years post injury.^18^ Hospital EDs are able to offer large populations of high-risk alcohol use populations who would be amenable to behavior modification intervention following an acute traumatic injury.^2,26,27^ ^28^

There have been no large-scale controlled studies in sub-saharan Africa evaluating the effectiveness of a BNI for alcohol. Given Sub-Saharan African patients have limited care-seeking behaviors and limited harmful alcohol use treatment options with a growing burden of alcohol, the unique opportunity to screen and intervene upon high proportions of high-risk alcohol behavior patients during a “teachable moment” in the ED in Tanzania, warrants an evaluation of an alcohol BNI effectiveness.

# Statement of the Problem:

Currently, we know that the injury patients who present to the KCMC Casualty Department have a high prevalence of harmful and hazardous drinking behaviors which might be amenable to intervention. While we have an intervention that has shown to be effective in high income settings, we have not had an intervention which is translated and validated for the Tanzanian environment to address high risk alcohol use.

# Rationale:

Given this need and potential intervention, this project will gather information in order to define the stigma, customs and cultures around alcohol use and will prepare and test an intervention for alcohol use in Tanzania.

# Broad Objectives:

Broad Objective 1: Describe the current perspectives on alcohol use

Broad Objectives 2: Develop a culturally appropriate intervention for alcohol use.

Broad Objectives 3: Conduct a pilot trial of the intervention.

# Specific Objectives:

Specific Objective 1: Define patient’s current perspectives on alcohol use.

Specific Objective 2: Define families’ and the public’s current perspectives on alcohol use.

Specific Objective 3: Define healthcare providers’ current perspectives on alcohol use.

Specific Objective 4: Translate, and iteratively improve a culturally-appropriate nurse-delivered brief negotiational interview (BNI) utilizing motivational interviewing, to be delivered in the KCMC Emergency Department.

Specific Objective 5: Validate measures which will be utilized to evaluate the intervention.

Specific Objective 6: Conduct a pilot trial of the intervention in order to validate study procedures.

Specific Objective 7: Conduct a pilot trial in order to evaluate the intervention acceptability.

Specific Objective 8: Conduct a pilot trial in order to evaluate the enrollment and retention rates.

# Methods:

**Setting***:* Moshi, a city in the Kilimanjaro region of Northern Tanzania with a population of 143,799, is home to Kilimanjaro Christian Medical Center (KCMC), the third largest hospital in the country and the referral hospital for northwestern Tanzania.^7^ KCMC is a regional training center for all types of healthcare workers and supports research as an integral part of its charter. Over the last 17 years, Duke and KCMC have developed a research infrastructure with proven success in NIH funded grants. Our current data from the KCMC Casualty (Emergency) Department suggests that 28% of all the patients who arrive to the Casualty Department for treatment of an injury consumed alcohol prior to their injury and therefore are ‘Hazardous drinkers’ who would be amenable to the proposed intervention ^29^.

**Research Team:** The research team who will conduct Aim1-3 is planned to be our current research team in Tanzania who have experience with research ethics, informed consent, data collection, data integrity, patient safety, focus groups and good clinical practices. All data collection nurses and data management personnel will have both their updated CITI certification, good clinical practices training updated as well as be trained in the protocol prior to any data collection. Study procedures and piloting of all questionnaires and study documents will take place prior to formal use of the documents.

## Aim 1: Identify patients, families, healthcare providers’ and the public’s current perspectives on alcohol use.

Study Design: We will conduct short surveys for KCMC ED patients, families and healthcare practitioners as well as focus groups for KCMC ED patients, families and community members to determine the 1) current perspectives of alcohol use including stigma associated with alcohol use, 2) harmful alcohol use behaviors and 3) barriers to intervention effectiveness.

**Focus Group Methodology:** Focus groups will be held in Swahili with patients or family members of patients of KCMC ED, as well as the KCMC Community Advisory Board (CAB) to better understand their perspective on: **availability of alcohol**, **stigma** associated with alcohol use or reporting behavior, **alcohol-related consequences**, and any **cultural barriers or culturally relevant solutions for** a successful intervention. Patients (or family members) will be identified in the KCMC ED waiting or treatment areas, after treatment or stabilization, and will be offered participation in the focus group. The focus groups will occur in a small quiet room near to the Casualty Department where patients or family members can freely discuss their thoughts and opinions. No members of the treatment team will be in the room and patients will be in different focus groups from their family members so that they can talk more freely about their thoughts on alcohol. We will attend a monthly CAB meeting after giving prior knowledge about the research activity in order to utilize this groups experience with research as well as their perspective as members of the public to conduct a focus group.

**Focus Group Data Collection:** Participants will take part in an informed consent process approved by the Duke and Tanzanian ethics committees before joining the focus group. Focus groups will be formed once 5-10 eligible interested participants are identified. Focus groups, led by trained research nurses, will be audiotaped and transcribed for formal qualitative analysis utilizing thematic analyses. For the CAB, we will announce our intention to conduct a focus group during their scheduled meeting. We will enroll participants after informed consent and will have about 40 members of the CAB available to share their experiences about alcohol. Transcriptions will occur within the days of the focus group and research nurse notes will be included in to these transcriptions about the content. We expect to host 8 focus groups or until saturation is reached.

**Focus Group Data Analysis:** After transcription, each of the focus groups will be translated and the final transcript will be discussed by the research nurses present in the focus group for content validation. Then a thematic analysis will be undertaken for each of the above main questions from the focus groups. All data will be analyzed with NVivo 10.0. All transcripts, audiotapes and other study documents will be kept for 6 years following conclusion of the data the conclusion of the study.

**Survey Methodology:** Based on prior and similar studies, we will conduct 3 separate cross-sectional surveys to determine attitudes and perceptions of alcohol use: 1) for patients >18 years of age who have suffered an injury; 2) for patients’ family members > 18 years of age; and 3) for healthcare professionals who are currently working clinically in the KCMC Emergency Department.^30^ ^31^ The surveys for patients will have a primary aim of determining at-risk and harmful alcohol use based on the AUDIT tool (See Appendix), an inclusion criterion for Aim 3. Additionally, the surveys for patients and families will determine **knowledge of drinking norms and alcohol use consequences, perspectives on drinking and on reducing alcohol use, perception of availability of alcohol, age of first alcohol ingestion and current treatment options** in the community for harmful alcohol use. A large portion of the survey will be adapted from the World Health Organization World Mental Health Composite International Diagnostic Interview. (WHO WMH-CIDI) Stigma associated with alcohol use will be determined with the alcohol adapted Perceived Devaluation Scale. (aaPDD) A validated tool for assessing alcohol related perceived stigma. **Surveys for healthcare practitioners will also query their role and responsibility in diagnosing and treating harmful alcohol use behavior.** The survey will include both short answer and open-ended questions to determine if there are more locally- relevant perceptions about alcohol use or treatment/ rehabilitation amongst patients with harmful alcohol use. After informed consent, patients or family members will be administered the survey by trained bilingual research nurses verbally in the native language of Swahili in a quiet room near the KCMC ED. For healthcare practitioners, surveys will be self-administered in written format in the medical language, English at participants’ leisure.

Study Population: We will enroll at least 323 patients, 226 patient family members and 45 healthcare practitioners including nurses, advanced medical officers and physicians from the KCMC ED to participate in the surveys. Focus groups with patients or patient family members will have 5-10 participants per group, totaling 60 participants. All participants will be >18 years of age and be able to speak Swahili (patients or families) or English (healthcare practitioners). We will exclude patients with inability to respond to the survey due to the severity of their illness or injury.

### Inclusion/ Exclusion Criteria:

Patients must be >17 years of age, be seeking care at KCMC for an acute (<6 hours) injury, be clinically sober at the time of enrollment, be medically stable, be able to communicate in Swahili or English and consent to participate. Patients will be excluded from enrollment if they are medically unstable or have a deteriorating condition, are too critically ill to participate, do not speak English or Swahili, are <18 years of age, are presenting for non-injury related complaints, are presenting or recheck or a second visit for their injury or are presenting after 6 hours after their injury, or do not consent to be enrolled.

Family members must be a family member of a patient who is able to be enrolled in the study who also agrees to participate, speaks English or Swahili, and feels like he/she knows the patient well enough to give perspectives on his/her alcohol use. For CAB members, we will enroll all interested participants who are >17 years of age and are present at the CAB meeting when we conduct the focus group discussion.

| All ED Pt/wk | Injury Patients/ wk, | Potential Enrollment per week | # weeks enrolling | Potentially Enrolled Patients |
| --- | --- | --- | --- | --- |
| Injury Pts ^1,2,3^ | 120 | 49 | 16 | 778 |
| Family Members ^1,2,3,4^ | 120 | 34 | 16 | 544 |
|  | Total ED Personnel | Anticipated Enrollment |  |  |
| HealthCare Professionals ^2^ | 50 | 45 |  | 45 |
| Community Advisory Board | 60 | 40 |  | 40 |
| **Table 4: Potential Survey Enrollment**: ^1^50% during enrollment hours, ^2^10% refusal, ^3^10% Lost to Follow up, ^4^70% with family member | | | | |

Healthcare practitioners who work in the Casualty Department/Emergency Department at KCMC and have patient contact are eligible to be enrolled.

Sample Size Calculation: For the injury patient surveys, sample size will be calculated to determine the proportion of harmful alcohol use based upon the AUDIT tool, (see Appendix) which will be an inclusion criterion for Aim 3. A sample size of 323 injury patients will allow us to estimate our anticipated AUDIT positive (>8) proportion of patients anticipated at 30% to within +/- 5% (95% confidence interval). However, given the patient volume, we likely will recruit additional patients. Surveys will be conducted for 16-24 weeks. Conservative estimates of patients who could be enrolled are seen in Table 4.

For family member surveys, our goal is to get 1 family member per patient yet recognizing that approximately 30% of patients may have no accompanying family members. Thus, our sample of family members is estimated to be at least 226 family members. As for the healthcare practitioners, there are 50 professionals affiliated or connected to the emergency department, considering a 10% loss due to rotation or annual leave, our aim is to get at least 45 practitioners included in the survey.

Data Collection: Surveys will be verbally administered to patients and families due to variable literacy rates. Surveys will be self-administered by healthcare providers in the standard medical language of English or Swahili. Focus groups will be conducted, once weekly until saturation, and will be audiotaped, transcribed, and translated. They will focus on the **prevalence of harmful and hazardous alcohol use**, the **cultural acceptance of alcohol**, **moderators of alcohol use**, and **current perspectives on the feasibility and potential impact of a brief intervention**.

Data Analysis: Survey results will be analyzed through a range of descriptive and analytical techniques. Pertinent outcome variables are expected to be dichotomous. (e.g., hazardous drinking, care-seeking behavior, injury outcomes). Then, for all three surveys (patients, family and healthcare providers) basic descriptive associations of sociodemographic characteristics and binary outcomes (when applicable) will be examined using chi-square tests, and logistic regression analyses. Our goal is to determine patterns of associations between sociodemographic variables, harmful alcohol use and related injury, and attitudes and practices towards alcohol use. Open-ended survey items and focus group data will be analyzed into response categories using nVivo. Latent Semantic Analysis and Sentiment Analysis will be used to investigate latent concepts or positive/negative tones behind the qualitative answers.

## Aim 2: Develop a culturally- and linguistically-appropriate, nurse-delivered BNI; validate measures to test its effectiveness

Summary: We will adapt, translate, and validate a BNI based on its content, adherence to the FRAMES platform of motivational interviewing, and face validity. In preparation for our pragmatic BNI trial we will also validate the evaluation tools of BNI effectiveness (AUDIT, DrInC, IBC, in C5.3) in Swahili. (See Figure 3)

| **Outcome Scales** | Internationally Validation | Tanzanian Validation |
| --- | --- | --- |
| Alcohol Use Disorder Identification Test (AUDIT) | Yes ^32,33^ | No |
| Drinker Inventory of Consequences | No | No |
| **Quantity/Frequency** | N/A | N/A |
| Injury Behavior Checklist | No | No |
| Table 6: Outcome Scales | | |

**Creation/adaptation of the BNI:**

| **FRAMES Model** | |
| --- | --- |
| **Feedback** | Give feedback on the risks and negative consequences of substance use.  Seek the client's reaction and listen |
| **Responsibility** | Emphasize the individual’s responsibility for making his/ her own decision about his/her alcohol use |
| **Advice** | Give straightforward advice on modifying alcohol use |
| **Menu of options** | Give menu of options to choose from, fostering the client's involvement in decision-making |
| **Empathy** | Be empathetic, respectful and non-judgmental. |
| **Self-Efficacy** | Express optimism about his/her ability to modify his/her substance abuse. |
| Table 5: FRAMES Model | |

Translation: First, we will create the BNI script based on US guidelines then, translate and back-translate the BNI script. We will use experienced local translators with knowledge of the local language to ensure correct terminology. After individual translations, a group assessment of the translation will be performed by our research nurses and project coordinator to ensure comprehension and appropriate word selection. We will also conduct 2-4 focus groups with 5-10 healthcare professionals and community members each in order to choose the appropriate translation of key important terminology for the intervention. (‘alcohol’, ‘alcoholic’, ‘motivate’ etc) Similarly, we will seek their opinions on some specific intervention details and their ‘cultural translation.’ For instance, we will ask if data from Tanzania about current drinking behaviors and drinking levels which are associated with increased injury or complications will be more motivating to patients than international data.

Content Validity: Next, consistency between the translations and content validity of the script will be verified; a panel of local healthcare practitioners will observe the intervention administered to a volunteer research nurse and will evaluate its content validity/coherence by 3 domains

(language, clarity, relevance/ importance). Suggestions for improvements will be sought from this panel with iterations of the BNI re-tested.

Adherence to FRAMES: This panel will then evaluate the BNI script’s adherence to the FRAMES model for motivational interviewing in order to develop the final version of the script (Table 5, ie, did the script give the patient feedback, did it empower the patient to choose to reduce drinking?).

Face Validity: Finally, after practicing and piloting the intervention on health practitioner volunteers, we will perform face validity testing and inter-rater agreement by having trained expert observers assess the intervention using the BNI Adherence Scale. ^34^ This scale has been used in other BNI assessment studies in order to gauge provider adherence to BNI protocol. Individual research nurse provider will receive feedback in order to improve BNI administration.

Creation of a SMS based test-based booster for the BNI

Step 1: We will **create the SMS Content** by compiling evidence from the literature about the specific theoretical framework for behavior change that we wish to utilize. We will use expert opinion, behavior change frameworks and any available examples of SMS-based alcohol related harm reduction strategies to create potential SMS content.

Step 2: We will **Translate/ Back translate and culturally adapt** the SMS content. After the content is created in step 1, we will perform an independent translation and back translation as well as a consensus translation meeting in order to ensure language translation, cultural appropriateness and content validity. We will consult with local physicians, researchers or health care workers in order to provide support for the clarity, pertinence to the theoretical domain, cultural pertinence and rank their preference of SMS content.

Step 3: We will ensure **cultural adaptation** through a cognitive interview of with less than 30 patients. Based on feedback from these interviews, SMS content will be further adapted before advancing to the next steps.

Step 4: Next, we will **Rank content** importance and ability to affect behavior change. By enrolling 50 patients we will have patients provide feedback on the clarity, rank his/her preferred or optimal SMS content, and their perception of how much each statement would likely change his/her behavior.

Step 5: Next, **SMS Ranking** will be performed so up to 50 injury patients will receive SMS content and will receive 2 questions per SMS text content requesting a response of a likert scale on how clear the overall texts are and how likely this SMS would change his/her behavior.

| **Step 1** | **Content Creation** | No enrollment |
| --- | --- | --- |
| **Step 2** | **Translation/Back Translation**  **Translation Meeting**  **Expert Opinion** | Research Team  Research Team  15 local experts |
| **Step 3** | **Cultural adaptation** | Enroll 30 patients for a cognitive interview |
| **Step 4** | **Rank Content** | Enroll 50 patients for a short survey |
| **Step 5** | **SMS Ranking** | Enroll 50 patients for a SMS based survey |

### Validation of Measures:

Outcome Scales: The outcome measures to evaluate the BNI include Alcohol Use Disorder Identification Test (AUDIT, See Appendix)^32^ ^35^, the Drinker Inventory of Consequences (DrInC)^32^ ^36^, drinking quantity and frequency data, and the Injury Behavior Checklist (IBC) as seen in Table 6.^37^ ^38^ ^39,40^ The AUDIT is a 10-item self-reported scale [range 0-40], a score of 8 or above has a 85% sensitivity and 89% specificity, measuring harmful or at-risk drinking.^36,41^ A score of at least 8,will be used to determine eligibility as scores of 8-15 are most appropriate to simple advice interventions.^35^ The DrInC is 45-item self-report questionnaire on negative consequences of drinking including physical, interpersonal, intrapersonal, social responsibility, and impulse control (See Appendix).^42^ The Injury Behavior Checklist (IBC) adapted from the Adolescent Health Status Instrument, was revised for adults and allows patients to record the number of times they suffered any of 18 injuries in the past year.^37^ ^38^ ^39^ This combination of scales has been used in a prior RCT testing the effectiveness of a BNI on re-injury rates.^40^

Validation of Scales: These measures have not been validated in a Swahili speaking KCMC patient population. Therefore we will perform validation and reliability testing. A sample of 300 subjects from general population and patients will be selected for the validation process. This sample size is determined by the number of items to be validated in each instrument (ie, DrInC has 45 items), we opted to recruit the suggested 5-10 subjects per questionnaire item as suggested in the literature.^43^ Scales (AUDIT, DrInC, IBC) will be administered to 300 subjects from the general population and patients in order to assess their psychometric properties and assure adequate cross-cultural adaptation and validity. Given the 9 months for enrollment for this portion of project, we anticipate that these numbers can be easily obtained from visitors to the hospital grounds. We will evaluate internal validity (factor analysis, content, convergent and discriminant validity), external validity that is the ability of the test to evaluate what it is supposed to measure (concurrent validity) and reliability (internal consistency). Based on preliminary enrollment, and finding a few comprehension challenges in our validation phase, we will need to enroll more patients at 500 patients in order to ensure appropriate validation.

Aim 3: Conduct a pilot trial of the intervention. Feasibility of 1) study procedures, 2) patient acceptability of the intervention and trial, and 3) patient enrollment/retention will be tested in a pilot RCT.

Introduction and Rationale: To test the intervention’s effectiveness for at risk and harmful alcohol use reduction, an adequately powered pragmatic clinical trial with maximum external validity is the next step to evaluate this intervention.^44^ To prepare for this clinical trial, a

feasibility trial should be conducted prior to a large scale trial, as the most essential pre-requisite of a trial enhancing the likelihood of success of a RCT.^45^

Research Design: This will be a pilot 4, 8 block randomized feasibility and acceptability trial.

Participants and Recruitment: We will prospectively enroll (n=75, across 4 arms) patients who present to the KCMC ED for care of acute injuries and meet inclusion and exclusion criteria: >18 years of age, be clinically sober at the time of enrollment, have capacity to give informed consent, converse in the local language Swahili or in English, and have either 1) reported ingesting alcohol in the 6 hours prior to injury, 2) have a positive breathalyzer test, or 3) have an Alcohol Use Disorder Identification Test with a score of ≥8. Our trained research nurses will describe the intervention as a health assessment, and eligible interested participants will be enrolled, provide informed consent and a randomization packet chosen. They will then administer patient demographic, injury, alcohol use surveys and the intervention if appropriate all lasting less than 45 minutes.

**Figure 1: Pragmatic Randomized Adaprtive Clinical Trial Design**


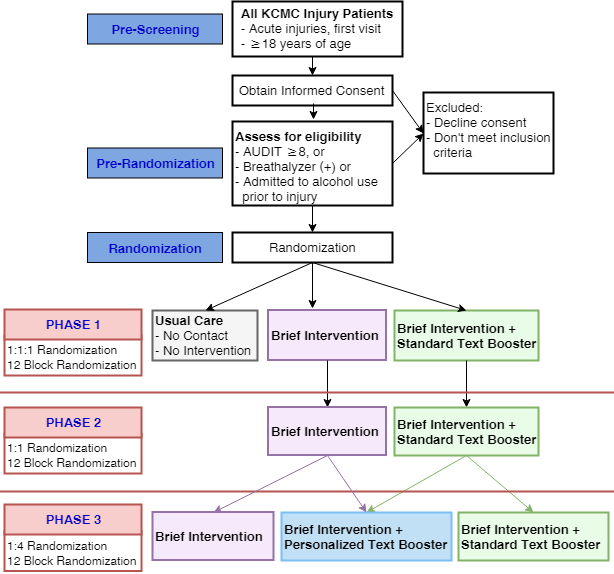


Follow up Procedures: All groups will provide two phone numbers where they can be reached in order to facilitate follow up. All participants will be contacted at 6 weeks, 3 months, and 6-months post-discharge for follow-up survey administration. Surveys are expected to be less than 20 minutes. All patients will be contacted by text message at the conclusion of the study to inform them about availability of the results of the study.

| **Study Procedures** | Protocol Deviation Event Analysis | Intervention Fidelity: BNI Assessment Observations | Survey Time |
| --- | --- | --- | --- |
| **Acceptability** | Questionnaires | | |
| **Recruitment & Retention** | **Enrollment Rate**  **Retention Rate** | Enrollment/Retention Failure Event Analysis | |
| Table 7: Feasibility Trial Outcomes | | | |

Feasibility Trial Outcomes**:** We will conduct a feasibility assessment subjectively through self-assessments as well as objectively through Event Analyses, understanding causes of deviations from protocol, Intervention Observations and Questionnaires.

Study Procedures: To evaluate the study procedures, we will evaluate any protocol deviation events, and compare length of survey times. We will also perform assessments of intervention fidelity through real-time observations of BNI administration using the BNI Assessment Scale similar to the procedure in Aim 2.

Intervention and Trial Acceptability: We will assess acceptability of participation in trial and intervention through questions after the final follow up timepoint for both the research team as well as participants. Assessment about the length of surveys, method of follow-up, number of follow-up calls,

Enrollment and Retention: The primary outcome measure will be recruitment and retention rates. Secondarily, we will qualitatively assess the inclusion and exclusion criteria, reasons for non-enrollment or non-retention, to the extent possible. Finally, follow-up questions about remuneration procedures in order to improve enrollment or retention will be administered after enrollment or on the completion of follow up.

Sample Size**:** In total, 75 participants will be recruited (randomized across 4 arms). This sample size is based on what is needed to evaluate study process fidelity, intervention acceptability, and recruitment and retention assessment. This sample size will allow us to estimate our desired minimal recruitment rate of 30% to within +/- 11% (95% confidence interval) and to estimate our desired retention rate of 80% within +/- 10%. Further, it is anticipated to provide sufficient opportunity to uncover significant acceptability concerns. We are not proposing an adequately powered study nor an effect size calculation as pilot studies may not accurately represent effect sizes of the main study. ^46^

### Ethical Considerations

**Subject Recruitment and Compensation**

For objective 1, all healthcare workers at KCMC Casualty Department will be offered enrollment in in the study. The only cost to participants is the time spent performing the survey. The participants will not be compensated for their time for this study. For objective 2, a snowball method of sampling key informants will be utilized to recruit subjects for enrollment in the key informant interview. Participants will not be compensated for their time spent completing the survey.

**Consent Process**

For patients and patients’ family members we will be utilizing a written informed consent. Participants will be provided with a description of the study objectives and procedures, along with information on data safety. Agreement to participate in the survey will serve as consent to participate in the study.

### Limitations of the study

Limitations of the study should be taken into account when planning for the project. This project is a multi-step project planned to describe both the general atmosphere around drinking alcohol as well as the current culture and stigma in Tanzania. As this employs both qualitative and quantitative methods across a broad area, there could be some limitations to the depth of understanding of a portion of the culture of drinking in Tanzania. As we are not making this a representative sample of the whole region of Tanzania just for Moshi, the generalizability of the qualitative data is limited and a repeat study should be performed in order to generalize our results to neighboring regions. Tools used in this study are the best available tools used currently in high income settings but their translation and adoption to the Tanzanian setting will require frequent assessment and an interative process of improvement to continue to use them and ensure their successful utility. While we have established sample sizes for both the qualitative, the validation and the pilot study based on the literature and current alcohol data from Tanzania these sample sizes might be unable to predict smaller subgroup findings. It is for this reason that we are conducting this pilot trial in order to gain a better understanding of the needed sample size for a larger trial.

**Dissemination and Publications:**

Dissemination of results will occur for both the academic literature in the form of international presentations and research manuscripts as well as the clinical environment at KCMC. We will offer to KCMC leadership to discuss our results during the weekly Clinical Case Conference in order to educate KCMC Clinicians. Similarly, presentations in the Casualty Department will take place for KCMC Casualty Department staff and all healthcare providers. We also hope to share these results with local mental health care providers and health policy officials in order to understand the burden of disease suffered by our patients in hopes of planning for the needs of health care professionals for these patients.

# References:
