# Supplementary material for: Development of culturally-appropriate text message booster content to follow a brief intervention focused on reducing alcohol related harms for injury patients in Moshi, Tanzania
Source: PLOS Glob Public Health. 2024 Jul 25;4(7):e0002717. doi: 10.1371/journal.pgph.0002717 (PMC11271911; doi:10.1371/journal.pgph.0002717)
Supplement: Attachment — Submitted filename: Reviewers Comments.docx [file pgph.0002717.s006.docx]

**REVIEWER’S COMMENTS**

**General comments:**

Thank you for the opportunity and honor given to me to review this study. The study is important for addressing the issue of alcohol use in the country. The manuscript is well-written. However, it may benefit more from language and typographical edits.

**Abstract**

It is well-written.

**Introduction**

The way the word ‘Swahili’ has been used in the sentence in line 96 needs revision

**Methods**

- The process followed to develop and validate the messages is clearly explained.
- Revise line 145 to read ‘self-awareness, goal setting, and motivation.’
- Revisit lines 190 to 192 and edit the text for clarity, especially regarding how parts of the sentence are connected. The same applies to the sentence in lines 192 to 194.
- It's not clear in line 193 why authors decided to assess the “potential impact of one of 10 text messages” while they developed 29 messages.
- It's not clear what the authors meant by ‘consent clarity’ in line 193. I suppose they meant ‘content clarity.’

**Results**

- Revise the heading in line 220; there are some repetitions of words.
- I suggest authors cross-check the text messages included in Table 1. For instance, in the Swahili text message "Kunywa kiasi chochote cha pombe kabla ya kuendesha gari au pikipiki imeonesha kuongeza hatari yako ya kuumia barabara. Kuwa salama, usilewe”, ‘kuumia barabara’ does not appear to be a proper translation of ‘having a road traffic injury.’ Similarly, I have problems taking ‘madhara ya pombe’ to mean ‘alcohol-related consequences”. I suggest that, in addition to expert judges used, authors give the developed text messages to a Swahili and English language expert for an additional and final review.
- The sentence in lines 264 to 266 is confusing; it needs revision for clarity. Moreover, authors need to cross-check whether what they have stated in the subsequent sentence (line 267) is valid.

**Discussion**

- Line 283 needs a typographical error correction (citation position).
- In the study limitation, it might be helpful to mention that the authors did not include an English–Swahili language expert, as stated above, in addition to using expert judges.
