# Supplementary material for: Development of culturally-appropriate text message booster content to follow a brief intervention focused on reducing alcohol related harms for injury patients in Moshi, Tanzania
Source: PLOS Glob Public Health. 2024 Jul 25;4(7):e0002717. doi: 10.1371/journal.pgph.0002717 (PMC11271911; doi:10.1371/journal.pgph.0002717)
Supplement: Attachment — Submitted filename: renamed_e1c8c.docx [file pgph.0002717.s007.docx]

Kathleen Bachynski, PhD, MPH
Academic Editor
PLOS Global Public Health

Dr. Bachynski:

We appreciate your valuable feedback on our paper. We have revised the manuscript, incorporating the insightful comments and suggestions provided by both yourself and the reviewers. Please find responses to each comment below:

**REVIEWER #1**

| **Comments and suggestions** | **Answer** |
| --- | --- |
| The way the word ‘Swahili’ has been used in the sentence in line 96 needs revision. | Thank you for your comment. It was altered in the text for clarity. |
| Revise line 145 to read ‘self-awareness, goal setting, and motivation.’ | This has been altered in the text. |
| Revisit lines 190 to 192 and edit the text for clarity, especially regarding how parts of the sentence are connected. The same applies to the sentence in lines 192 to 194. | This section was revised to improve clarity. |
| It's not clear in line 193 why authors decided to assess the “potential impact of one of 10 text messages” while they developed 29 messages | Thank you for your comment. We didn't want the patients to review all 29 messages. Therefore, we divided the messages so that each participant reviewed up to 10 messages. We clarified this in the methods. |
| It's not clear what the authors meant by ‘consent clarity’ in line 193. I suppose they meant ‘content clarity.’ | This line was revised to improve clarity. |
| Revise the heading in line 220; there are some repetitions of words. | The text was altered. |
| I suggest authors cross-check the text messages included in Table 1. For instance, in the Swahili text message "Kunywa kiasi chochote cha pombe kabla ya kuendesha gari au pikipiki imeonesha kuongeza hatari yako ya kuumia barabara. Kuwa salama, usilewe”, ‘kuumia barabara’ does not appear to be a proper translation of ‘having a road traffic injury.’ Similarly, I have problems taking ‘madhara ya pombe’ to mean ‘alcohol-related consequences”. I suggest that, in addition to expert judges used, authors give the developed text messages to a Swahili and English language expert for an additional and final review. | We appreciate your remark. We had included expert local judges to review the messages. |
| The sentence in lines 264 to 266 is confusing; it needs revision for clarity. Moreover, authors need to cross-check whether what they have stated in the subsequent sentence (line 267) is valid. | Thank you for your feedback. The text was altered to improve clarity. |
| Line 283 needs a typographical error correction (citation position). | Altered from ‘. (30)’ to ‘(30).’ |
| In the study limitation, it might be helpful to mention that the authors did not include an English–Swahili language expert, as stated above, in addition to using expert judges. | We included this statement on the limitations. |

**REVIEWER #2**

| **Comments and suggestions** | **Response** |
| --- | --- |
| The title seems very long to me and I do not understand it well. I would remove ‘to follow a brief intervention’ or use a different wording | Thank you for your suggestion. We have made the necessary changes to improve clarity. |
| The abstract in itself does not give me a clear view of the study. Reading it as a standalone abstract raises many questions. It is not clear to me what is meant by ‘culturally appropriate’ | We appreciate the insightful feedback provided by the reviewer. By 'culturally appropriate,' we mean that the messages should resonate with the cultural context of the recipients, ensuring that they are relatable, understandable, and acceptable within their cultural framework. This involves adapting the content to match local customs, beliefs, and communication methods, ultimately improving the impact of the intervention. |
| I would expect more on the BNI. The sentence ‘Creation of text message boosters for an ED-based intervention expands the window of opportunity for alcohol use reduction in this high-risk population.’ does not add to the abstract as it is not clear how. Also, the abbreviation ED is not written out anywhere. | We appreciate the suggestion. The text was revised and changed. The long-form has been added as an abbreviation at the abstract, as well as the first use in the body of the manuscript. |
| I am not sure how the methods lead to cultural appropriateness. There is no explanation on who was involved in the methods and how analyses was done. | We value your input. The text has been adjusted to improve understanding. |
| ‘yet only 29 text messages were successfully translated and adapted’. It is not clear why only 29. Were the others simply not done? Or were they not appropriate? If so, who decided on that? | Thank you for your feedback. Adjustments have been made to improve comprehension. |
| ‘We developed culturally appropriate text message boosters in Swahili for injury patients in Tanzania coupled with a BNI for alcohol use reduction.’ This abstract is not about developing the BNI. I would leave that out. | We appreciate your suggestion. The changes were made to increase clarity. |
| The sentence ‘We found it important to evaluate content validation for interventions and measurement tools because the intended message can often be lost in translation.’ Seems overdone. In this abstract there is nothing about measurement tools. | Reference to measurement tools was removed. |
| Line 75: ‘Further, the Kilimanjaro region also exhibits a higher prevalence of alcohol consumption compared to the rest of Northern Tanzania, potentially due to different reporting behaviors, socioeconomic status, and cultural practices’. I would expect an explanation on how. | The text was altered to improve comprehension. |
| Line 77: ED is used, but there is no explanation on what ED is | We appreciate the suggestion. The long-form has been added at first use in the abstract, as well as the first use in the body of the manuscript. |
| mHealth: the term seems to be ‘old-fashioned’. I would change it to a more modern term such as digital health. | Thank you for this suggestion. This term has been updated accordingly. |
| Line 91: Text Message should be Text messages | The line has been changed. |
| Page 4, paragraph 1: more information is needed on BNI | We appreciate your feedback. The paragraph was improved to explain the BNI better. |
| Page 4, paragraph 2: more information is needed on what a text message booster entails | Thank you for the feedback, we included information to the paragraph. |
| This study is mentioned to be a mixed-methods study, but I do not see any quantification of results. | The study was a two-phase qualitative study. It was corrected in the text. |
| Line 113: ‘Our pilot study registration number is NCT02828267, and our ongoing clinical trial is 114 registered with ClinicalTrials.gov as NCT04535011.’ The described study is not a pilot study, neither a trial. How does the described study fit with these two mentioned studies? | A sentence was added in this section to clarify that the described study was one component of the larger protocols to pilot and trial the interventions. |
| Line 111: It is not NIMR who approves the proposal, it is the NATHREC based at NIMR. Also, for KCMC, it is CRERC who approves the proposal. | Details were added to clarify the names of the ethics committees. |
| Line 120: Reference 17 is according to the title of that publication not about education, but purely about data collection in a very specific group of people. Other studies in Moshi, among patients, have shown much higher percentages. | Thank you for your suggestion. Adjustments have been made for better understanding with different references. |
| Line 124: Are the text messages for the BNI or meant as a booster? | Details were added to clarify that the text messages were separate, but intended to boost effectiveness of the BNI. |
| In the abstract, it looks as if messages were found to be themed on self-awareness, goal setting and motivation. But in the methods the themes part of the selection criteria. Therefore, in the abstract, it should also be under methodology and not under results. | The text was improved in clarity. The selection of the three areas—self-awareness, goal setting, and motivation—stems from our experience in addressing the specific needs of this population; they encompassed necessary components of successful, sustainable behavior change, and represented areas Tanzanian health professions identified as critical to an alcohol harm reduction intervention in this setting. |
| Line 143: Why were members of the research team editing the message pool and not end-users (like those who drink alcohol and are ending in ED)? How are the research members representative? What are their functions/positions and why were they the ones working on that? | This information was added to the paragraph. Since end users don't speak English, it was necessary to guide them through the process in English to ensure they understood the goals. To accomplish this, we enlisted Research Assistants who are Tanzanian locals with experience working with the target population. |
| Line 149: What is the definition of cultural appropriate changes? | The text was changed to increase clarity. The change of the messages is to make sure the message reflects the context accurately. Use clear language that makes sense. The content provided is a high-level overview in English, meant to offer a general understanding, hence the involvement of three team members. |
| Page 7, 2nd paragraph: Why the judges were not lay end-users? I would expect that they are much more knowledgeable about understanding of messages than the experts. | Your feedback is greatly appreciated. The lay end-users don't speak English. We included local experts as judges for their comprehension of both languages and also theoretical knowledge and experience working with this population. |
| Line 168: ‘The surveys contained…’ Which surveys? Were surveys used to evaluate the content? If that is the case, it should be added to the sentence of line 166. Also, were the questions open or closed-ended? | We appreciate your suggestion. Adjustments have been made to enhance clarity. We specified that we included the qualitative results and analysis. While we anticipated doing a survey on the content validity, we focused on open ended responses to receive suggestions for improvement. Thus, we edited the text to clearly state it was a qualitative evaluation of an open-ended assessment. |
| Line 188: What is the ‘planned Kilimanjaro Clinical Research Institute (KCRI) Short Message Service (SMS) System?’ How does it work? After reading the referred publication of Pima et al., I assume it is the same system? Then it could be described as such. | Thank you for your comment. This part of the manuscript referred to part of the feasibility assessment of the system itself. We decided to remove this portion because this study focused on the content development and not the system itself. |
| Line 202: What is the definition of cultural adaptation problems? | Thank you for your input. The manuscript has been amended with this information earlier in the methods section. The challenges in translating the messages lie in making them cohesive with the same meaning in Swahili, as many words do not translate as expected according to the original English message. |
| What were the ethical considerations? Was informed consent used for all steps? | We appreciate your question. Yes, there was consent at every step from all the experts. |
| Line 212: It is not clear how subthemes were identified. Could this be elaborated on in the methods? | The text was altered to improve clarity. We specified that sub-themes were originated from a content analysis. |
| Table 1: The table shows personalized messages, but it is not clear how these were created and why. | Text was added to improve the clarity. |
| I do not completely agree that cheeseburgers are not part of TZ culture. Cheeseburgers can be found more and more on different menus, including at local places. | The text has been changed to improve the understanding of the interpretation of the example used. We also apologize for making it sound as cheeseburgers were not part of the Tz culture. We understand that the caloric interpretation is not as relevant to the culture in Tz as it is in the US and other Western countries. |
| In the methods, it is described that three members of the research team worked on cultural appropriateness. How was this done in an objective way and what were the results obtained from this step? | Thank you for your feedback. The text has been improved for better understanding. W described that they evaluated the content independently and in parallel and then reached a consensus on changes needed. We summarized the changes needed along with the other content changes that were observed. |
| The paragraph from line 226 is very informative and interesting. Though reading, this implies that some kind of qualitative data was collected leading to these results though the first sentence mentions that these results came from a survey. | Thank you for your feedback. It was a data collection process. The text was changed. |
| Line 250: Why were only 29 completed in the process? What happened to the other messages? | Not all messages made sense in the cultural context due to behaviors and habits that are not as common in Tanzania. |
| Line 260: The SMS system was not working… I wonder if it makes sense to mention this system at all in the manuscript. What does it still add to the manuscript while it wasn't used? If left in, a clear explanation is needed on why it didn’t work as despite of the sent SMSs of the BNI working, it will also raise questions about the feasibility of that. | Thank you for your feedback. We considered it and removed this part of the text. |
| Line 295: Sentence starting with ‘For instance,….’ seems to be grammatically incorrect. | This sentence was modified to improve clarity, though we note that beginning the sentence with this phrasing is, indeed, grammatically correct, and has been used in the journal. |
| The fact that the SMS system for data collection was not working seems surprising to me. Reading the publication from Pima et al (that has been referred to several times) that seems to come from the same institute (KCRI?), there were no problems with such data collection. Again, I wonder to what extent mentioning this adds to the current manuscript. | This was removed from the manuscript. |

**REVIEWER #3**

| **Comments and suggestions** | **Response** |
| --- | --- |
| The process of testing the messages with prospective patients is under reported, and as there appeared to be major technological issues with this, this omission is a significant problem. Using the CONSORT-eHealth ([https://pubmed.ncbi.nlm.nih.gov/22209829/](https://urldefense.com/v3/__https:/pubmed.ncbi.nlm.nih.gov/22209829/__;!!OToaGQ!tsjEVPF_ous9UfNXZyd6AakXPgXfCnM-rn25z2VV0lsm5u3qsHQGfCXNxioo7FvsOi7RguplL0SeV4RE03k-tQ$)) or mERA guidelines ([https://www.bmj.com/content/352/bmj.i1174?etoc=](https://urldefense.com/v3/__https:/www.bmj.com/content/352/bmj.i1174?etoc=__;!!OToaGQ!tsjEVPF_ous9UfNXZyd6AakXPgXfCnM-rn25z2VV0lsm5u3qsHQGfCXNxioo7FvsOi7RguplL0SeV4RXcOtw2w$)) would improve the reporting of the test interventions used to test messages. | We appreciate your feedback. We removed the prospective testing due to an issue with the data collection system. |
| It is unclear from the writing if any feedback was collected from patient. On page 12, line 265, it states that the research team was able to discuss each text message, but the rest of the manuscript does not report any notes or analysis of these discussions. If the discussions occurred, some report of the methods used to gather information should be reported, even if informal. If this is a type, it should be clarified, adn the lack of patient feedback should be added to the discussion and limitations. | We clarified this topic. We summarized all the changes needed on Table 2. We did not receive feedback on edits to the messages from the 15 participants included in the study at the pilot phase. We then reported that the patients found the messages to be clear and contextually relevant. |
| The lack of patient or community feedback on the messages is a potentially a fatal flaw; this adaptation of messages from high income countries to LMIC did include local clinicians, but the feedback from community members or patients is necessary prior to implementation; with out this, the potential benefit maybe substantially delayed. | We apologize for not making this clearer. We did not receive any new additional feedback from the community (patients) at the piloting phase, through our cognitive interviews. |
