# Supplementary material for: Development of culturally-appropriate text message booster content to follow a brief intervention focused on reducing alcohol related harms for injury patients in Moshi, Tanzania
Source: PLOS Glob Public Health. 2024 Jul 25;4(7):e0002717. doi: 10.1371/journal.pgph.0002717 (PMC11271911; doi:10.1371/journal.pgph.0002717)
Supplement: Attachment — Submitted filename: Reviewers Comments.docx [file pgph.0002717.s008.docx]

**Reviewer’s Comments**

The authors have worked well on most of the comments I provided. Just a few more comments in the following sections:

**General comments**

1. Typographical errors are still in the manuscript. Authors should go through the entire manuscript to ensure such errors are corrected. For example:
   - Line 197 to 199 reads “Next, we enrolled 15 patients to receive these texts on their phones, and our research team solicited feedback on the content clarity, cultural appropriateness. They should add ‘and’ after the last coma.
   - The sentence in line 199 “Each patient evaluated up to 10 text messages, until al messages were” ‘al’ should be corrected
2. The clarity of the manuscript could be much improved if authors considered seeking assistance from an English editor.
